# Self-Reported Smell and Taste Disorders in the General Population: A Cross-Sectional Survey in Austria, Germany and Switzerland

**DOI:** 10.1007/s00405-026-10189-w

**Published:** 2026-03-28

**Authors:** Umar Rehman, Carl Philpott, Julian Hsieh, Christian A. Mueller, Basile N. Landis, Antje Welge-Luessen, Andreas Huber, Martin Sylvester Otte, Jens Peter Klussmann, Matt Lechner, Thomas Hummel

**Affiliations:** 1 Division of Surgery and Interventional Science, University College London, London, UK; 2 Norwich Medical School, University of East Anglia, Norwich, UK; 3 The Norfolk Smell & Taste Clinic, Norfolk & Waveney ENT Service, James Paget University Hospitals NHS Foundation Trust, Great Yarmouth, UK; 4 Department of Otorhinolaryngology-Head and Neck Surgery, Geneva University Hospitals, Geneva, 1205 Switzerland; 5 Department of Otorhinolaryngology, Head and Neck Surgery, Medical University of Vienna, Vienna, Austria; 6 Department of Otorhinolaryngology, Head and Neck Surgery, University Hospital Basel, Basel, Switzerland; 7 Tyrolean Federal Institute for Integrated Care, Tirol Kliniken GmbH, Innsbruck, Austria; 8 Medical Faculty, Department of Otorhinolaryngology, Head and Neck Surgery, University of Cologne, Cologne, Germany; 9 Center for Molecular Medicine Cologne (CMMC), Faculty of Medicine, University of Cologne, University Hospital Cologne, Cologne, Germany; 10 UCL Cancer Institute, London, UK; 11 Department of Otorhinolaryngology, Smell and Taste Clinic, Technische Universität Dresden, Dresden, Germany

**Keywords:** COVID-19, Prevalence, Quality of Life, Smell and Taste Disorders

## Abstract

**Purpose:**

To assess the prevalence, symptom profiles, and quality-of-life (QOL) impact of Smell and Taste Disorders (SATDs) across adult populations in Austria, Germany and Switzerland.

**Methods:**

A cross-sectional online survey was conducted in January 2025 among 2,340 non-institutionalised adults (≥ 18 years) residing in Austria (*n* = 1,023), Germany (*n* = 1,031), and Switzerland (*n* = 286). Participants were recruited via Dynata, an established online research panel whose members had consented to receive survey invitations. Respondents completed the culturally adapted and validated Smell-Qx questionnaire assessing SATDs, aetiology, and quality of life. Multivariable linear regression identified predictors of reduced QOL, measured using the Smell-Qx QoL item.

**Results:**

Overall, 40% (*n* = 945) of respondents reported at least one symptom, with olfactory intolerance (29%, *n* = 686), phantosmia (27%, *n* = 622), and parosmia (19%, *n* = 451) being the most common. Among those with SATDs, 62% reported unchanged or worsening symptoms since onset, and 41% reported decreased life satisfaction. An adjusted analyses showed: phantosmia (*p* < 0.001) and parosmia (*p* < 0.001) were the strongest independent predictors of reduced QoL.

**Conclusion:**

Qualitative symptoms, particularly parosmia and phantosmia, are prevalent and persistent in the post-COVID population and are strongly associated with diminished quality of life. Although the voluntary nature of survey participation is likely to favour selection bias, probably overrepresenting individuals with more persistent or severe symptoms, the present findings certainly reflect a shifting aetiology of SATDs and highlight the need for targeted treatments, structured care models, and multidisciplinary approaches to address this emerging public health burden.

## Background

Smell and taste disorders (SATDs) have emerged as important public health concerns, particularly following the onset of the COVID-19 pandemic [1–6]. These chemosensory functions are essential for food enjoyment, social interaction, personal safety, and mental wellbeing [7–9]. Impairments can lead to significant reductions in quality of life (QOL) and are associated with increased risks of depression, malnutrition, and social isolation [9].

Prior to the pandemic, sinonasal diseases were widely recognized as the most common cause of olfactory dysfunction in Central Europe [10]. A 2004 survey of otorhinolaryngology departments across Austria, Germany and Switzerland found that an estimated 79,000 patients were treated annually for olfactory disorders, with 72% of cases attributed to inflammatory diseases of the nose and paranasal sinuses. At that time, therapeutic strategies were largely centred around the use of corticosteroids, whilst smell training was less commonly used [10].

SARS-CoV-2 has been linked to SATDs, both during the acute phase of infection and, in some cases, persisting for prolonged periods beyond the initial illness [1, 3, 11]. Other causes of SATDs include, but are not limited to, non-COVID post-infectious causes, sinonasal disease, post-traumatic injury, neurological conditions, drug-related effects, congenital conditions, idiopathic, and iatrogenic causes [12]. Previous data from the UK indicates that 14.3% of the population reported experiencing smell loss since January 2020, corresponding to an estimated 9 million individuals affected during the COVID-19 pandemic. Similar prevalence rates have been observed in the USA, with broader estimates suggesting that up to 22% of the general population may be affected by olfactory disorders [1, 3, 13, 14]. However, comparable, population-level data from European countries is limited.

SATDs can be broadly classified into quantitative and qualitative types [12, 14, 15]. Quantitative disorders involve a reduced or complete loss of smell or taste perception, including symptoms such as hyposmia, anosmia, hypogeusia, and ageusia. Qualitative disorders refer to distortions in sensory perception. These include parosmia, where odours are perceived differently than usual, and phantosmia, which is the perception of an odour, often unpleasant, in the absence of any external stimulus. Olfactory intolerance, characterised by an abnormal sensitivity or aversion to everyday smells, is also included within this category. These classifications frequently overlap, and individuals may experience a combination of symptoms that persist or develop beyond the acute insult [12, 14, 15].

Although substantial attention has been paid to the relationship between SATDs and COVID-19 during the acute illness [3, 11, 16], few studies have examined the broader epidemiological impact in the general population or the extent to which symptom burden has changed since the pandemic. There is currently no large-scale study evaluating the prevalence of SATDs in Central Europe in the post-pandemic context. Understanding how the aetiological landscape has evolved is essential for informing diagnosis, treatment planning, and public health response.

## Objectives

The aim of this study was to assess the prevalence, symptom characteristics, and self-reported impact of SATDs among gender- and age-balanced adult populations in Austria, Germany, and Switzerland.

## Methods

### Study Design

A cross-sectional survey using a quota-balanced approach across age and sex was conducted in January 2025 using an online platform. This study was conducted and reported in accordance with the Strengthening the Reporting of Observational Studies in Epidemiology (STROBE) guidelines for cross-sectional studies.

## Setting

Participants were non-institutionalized adults (aged ≥ 18 years) residing in Austria, Germany, or Switzerland. Individuals were recruited (*n* = 3347) via Dynata (Shelton, Connecticut, USA) who have an online research panel, a pool of adults who have previously consented to receive survey invitations for research purposes [17]. Invitations were sent via email, with reminders issued to improve response rates. Stratified quota sampling was applied to ensure that the sample was demographically representative of each country’s adult population with respect to age, sex, and geographic region, based on national census data.

## Participants

Eligible participants were required to:

- Be aged 18 years or older;
- Be fluent in one of the target languages (German, French, or Italian);
- Reside in Austria, Germany, or Switzerland;
- Provide informed consent after reviewing a study information sheet.

## Variables

Data were collected using the Smell Qx, a validated, patient-reported outcome measure designed to comprehensively assess SATDs. The instrument includes 70 items spanning 11 domains: olfactory loss, parosmia, phantosmia, olfactory intolerance, gustation, symptom descriptors, aetiology, trigeminal symptoms, quality of life (QOL), and overall life satisfaction [18].

Quality of life related to SATDs was evaluated using a single global impact item, rated from 0 (“no problem”) to 5 (“as bad as it could be”). General life satisfaction was assessed with five items using a 7-point Likert scale (1 = “strongly disagree” to 7 = “strongly agree”), producing a total score range from 5 to 35 [18]. Complete responses were defined as participants who provided information on all primary symptom domains (Smell Loss, Parosmia, Phantosmia, Olfactory Intolerance, Taste Disturbance), age, sex, and SATDs-specific Quality of Life (QoL).

## Data sources and measurement

The Smell Qx was translated into German, French, and Italian following International Society for Pharmacoeconomics and Outcomes Research (ISPOR) and World Health Organisation (WHO) guidelines for cross-cultural adaptation of patient-reported outcome measures [19, 20]. This involved dual independent forward translation, reconciliation, backward translation by blinded linguists, expert panel review, and harmonization to ensure semantic and conceptual equivalence across all language versions.

## Statistical methods

Descriptive statistics were used to summarize demographic variables and symptom prevalence. Pearson’s chi-square tests were employed to compare prevalence across countries. To identify independent predictors of SATDs presence, multivariable logistic regression models were constructed, incorporating age, sex, country, smoking status, and other covariates. Prevalence estimates were extrapolated to national populations using 2025 population figures for Austria, Germany, and Switzerland, based on current demographic data [21].

Ordinal logistic regression was used to assess the relationship between the presence of SATDs and quality of life, assuming proportional odds. The global SATDs-QOL item was used as the dependent variable. All statistical analyses were performed using IBM SPSS Statistics for Windows, Version 27.0 (IBM Corp., Armonk, NY, USA), with significance defined as *p* < 0.05.

## Missing data

Missing data for primary variables were handled using a complete-case approach. Only participants with complete information on the variables included in each analysis were considered. Optional questionnaire items with partial responses were analysed separately using available cases. For ordinal logistic regression models assessing associations between SATDs and QoL, the proportional odds assumption was tested and met (Test of Parallel Lines, *p* > 0.05). Moreover, regression models were restricted to these complete cases.

## Results

### Participants

A total of 3,028 respondents submitted the survey, of whom 2,340 (69.9%) provided complete responses for all primary symptom domains, age, sex, and SATDs-specific quality of life (QoL), distributed across Austria (43.7%), Germany (44.1%), and Switzerland (12.2%) (Table 1). The proportion of complete responses by country was 79.8% for Austria, 75.0% for Germany, and 41.5% for Switzerland.

**Table 1:** Respondent Demographics

	Total (*N* = 2340)
**Age**	
18–24	239 (10.2%)
25–34	440 (18.8%)
35–44	420 (17.9%)
45–54	397 (17.0%)
55–64	425 (18.2%)
65–74	317 (13.5%)
75 or older	102 (4.4%)
**Gender**	
Female	1215 (51.9%)
Male	1121 (47.9%)
Non-binary	4 (0.2%)
**Marital status**	
Married	1076 (46.0%)
Separated or Divorced	268 (11.5%)
Single	996 (42.6%)
**Country**	
Austria	1023 (43.7%)
Germany	1031 (44.1%)
Switzerland	286 (12.2%)
**Smoking Status**	
Never smoked/chewed tobacco	1437 (61.4%)
Current smoker/chewing tobacco	393 (16.8%)
Ex smoker/previous tobacco chewing	510 (21.8%)
**Alcohol > 4 units per day**	
No	2028 (86.7%)
Yes	312 (13.3%)
**Education**	
Less than high school	805 (34.4%)
Completed high school	443 (18.9%)
Some college/university	265 (11.3%)
Completed Bachelors degree or equivalent	753 (32.2%)
Completed postgraduate degree or equivalent	74 (3.2%)

### Descriptive data

**Fig. 1:**
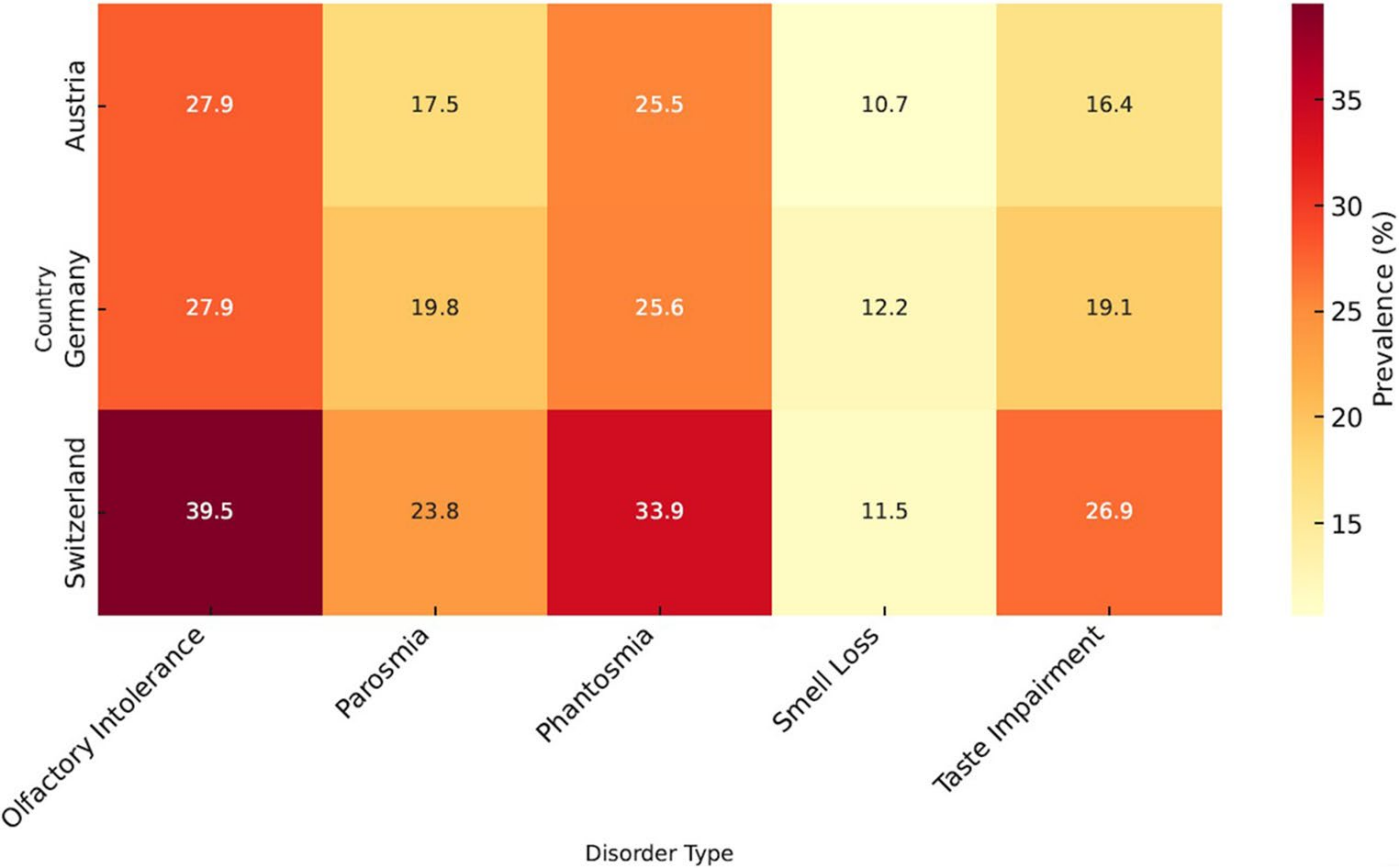
Heat Map of SATDs Prevalence by Country

Overall, 40.4% (*n* = 945) of respondents reported experiencing one or more SATDs. Olfactory intolerance was the most commonly reported symptom (*n* = 686, 29.3%), followed by phantosmia (*n* = 622, 26.6%) and parosmia (*n* = 451, 19.3%), with breakdowns by country shown in Table 2; Fig. 1.

**Table 2:** Prevalence of SATDs

	Austria	Germany	Switzerland	Total (*N* = 2340)
**Smell Loss**				
Yes	109 (10.7%)	126 (12.2%)	33 (11.5%)	268 (11.5%)
No	914 (89.3%)	905 (87.8%)	253 (88.5%)	2072 (88.5%)
**Parosmia**				
Yes	179 (17.5%)	204 (19.8%)	68 (23.8%)	451 (19.3%)
No	844 (82.5%)	827 (80.2%)	218 (76.2%)	1889 (80.7%)
**Phantosmia**				
Yes	261 (25.5%)	264 (25.6%)	97 (33.9%)	622 (26.6%)
No	762 (74.5%)	767 (74.4%)	189 (66.1%)	1718 (73.4%)
**Olfactory Intolerance**				
Yes	285 (27.9%)	288 (27.9%)	113 (39.5%)	686 (29.3%)
No	738 (72.1%)	743 (72.1%)	173 (60.5%)	1654 (70.7%)
**Taste Impairment**				
Yes	168 (16.4%)	197 (19.1%)	77 (26.9%)	442 (18.9%)
No	855 (83.6%)	834 (80.9%)	209 (73.1%)	1898 (81.1%)
**Number of SATDs**				
0	633 (61.9%)	613 (59.5%)	149 (52.1%)	1395 (59.6)
1	104 (10.2%)	121 (11.7%)	24 (8.4%)	249 (10.6%)
2	57 (5.6%)	54 (5.2%)	26 (9.1%)	137 (5.9%)
3	140 (13.7%)	135 (13.1%)	40 (14.0%)	315 (13.5%)
4	81 (7.9%)	95 (9.2%)	43 (15.0%)	219 (9.4%)
5	8 (0.8%)	13 (1.3%)	4 (1.4%)	25 (1.1%)

A total of 454 (48.0%) respondents provided information on the suspected aetiology of their symptoms; all (100%) indicated that symptoms began following an acute upper respiratory tract infection, such as a cold, influenza, or COVID-19.

Among those reporting SATDs (*n* = 945), 22.3% (*n* = 211) reported that symptoms had a moderate (3/5) to severe (4/5) impact, or described the problem as being “as bad as it could be” (5/5) in terms of quality of life. In total, 725 respondents provided data on life satisfaction. Among these, 40.6% (*n* = 294) reported being extremely dissatisfied, dissatisfied, or slightly dissatisfied with life due to their SATDs.

### Main results

#### Recovery of Symptoms

Participants were asked whether their symptoms had improved, worsened, or remained unchanged since onset. Across all three countries, 61.6% (*n* = 165) of respondents with smell loss reported no change or worsening of symptoms. This figure was 60.5% (*n* = 273) for parosmia and 48.4% (*n* = 301) for phantosmia. Similarly, 61.1% (*n* = 419) reported persistent or worsening olfactory intolerance, and 54.3% (*n* = 230) reported no improvement or worsening of “taste” loss. Full data are provided in Table 3.

**Table 3:** Recovery outcomes of SATDs by symptom type

Domain	Country	Total	Improved (*n*, %)	No Change (*n*, %)	Worsened (*n*, %)	Number of patients and % of total population with no change/worsening symptoms	Extrapolation to entire population (95% CI)**
Smell Loss	Austria	109	36 (33.0%)	64 (58.7%)	9 (8.3%)	73 (7.1%)	647,064 (547,662–746,466
	Germany	126	50 (39.7%)	57 (45.2%)	19 (15.1%)	76 (7.4%)	6,221,556 (5,703,239-6,739,873)
	Switzerland	33	17 (51.5%)	13 (39.4%)	3 (9.1%)	16 (5.6%)	502,175 (348,950 − 655,399)
	**Total**	**268**	**103 (38.4%)**	**134 (50.0%)**	**31 (11.6%)**	**165 (7.1%)**	
Parosmia	Austria	179	66 (36.9%)	108 (60.3%)	5 (2.8%)	113 (11.0%)	1,002,493 (901,216-1,103,770)
	Germany	204	82 (40.2%)	113 (55.4%)	9 (4.4%)	122 (11.8%)	9,920,859 (8,972,431 − 10,869,287)
	Switzerland	68	30 (44.1%)	32 (47.1%)	6 (8.8%)	38 (13.3%)	1,192,665 (947,295-1,438,035)
	**Total**	**451**	**178 (39.5%)**	**253 (56.1%)**	**20 (4.4%)**	**273 (11.7%)**	
Phantosmia	Austria	261	136 (52.1%)	124 (47.5%)	1 (0.4%)	125 (12.2%)	1,111,856 (999,899-1,223,813)
	Germany	264	154 (58.3%)	104 (39.4%)	6 (2.3%)	110 (10.7%)	8,996,033 (7,961,097 − 10,030,969)
	Switzerland	97	31 (32.0%)	57 (58.8%)	9 (9.3%)	66 (23.1%)	2,071,471 (1,547,017 − 2,595,926)
	**Total**	**622**	**321 (51.6%)**	**285 (45.8%)**	**16 (2.6%)**	**301 (12.9%)**	
Olfactory Intolerance	Austria	285	110 (38.6%)	160 (56.1%)	15 (5.3%)	175 (17.1%)	1,558,421 (1,411,318-1,705,524)
	Germany	288	125 (43.4%)	147 (51.0%)	16 (5.6%)	163 (15.8%)	13,282,862 (12,045, 413 − 14,520,311)
	Switzerland	113	32 (28.3%)	71 (62.8%)	10 (8.8%)	81 (28.3%)	2,519,841 (2,130,377-2,909,305)
	**Total**	**686**	**267 (38.9%)**	**378 (55.1%)**	**41 (6.0%)**	**419 (17.9%)**	
Taste	Austria*	149	64 (43.0%)	56 (37.6%)	29 (19.5%)	85 (8.3%)	756,427 (573,150–939,704)
	Germany	197	92 (46.7%)	65 (33.0%)	40 (20.3%)	105 (10.2%)	8,575,658 (7,924,894-9,226,423)
	Switzerland	77	37 (48.1%)	31 (40.3%)	9 (11.7%)	40 (14.0%)	1,255,437 (1,014,541-1,496,332)
	**Total**	**423**	**193 (45.6%)**	**152 (35.9%)**	**78 (18.4%)**	**230 (9.8%)**	

*19 participants did not respond to this item. ** Extrapolated population estimates were calculated by applying observed sample prevalence rates to national population figures. These estimates are illustrative only, reflecting potential scale, and should not be interpreted as precise epidemiologic counts, as they assume uniform prevalence and are based on a non-probabilistic, self-reported sample

As a proportion of the total sample (*N* = 2,340), 7.1% (*n* = 165) of participants reported persistent or worsening smell loss, 11.7% (*n* = 273) parosmia, 12.9% (*n* = 301) phantosmia, and 17.9% (*n* = 419) olfactory intolerance. Additionally, 9.8% (*n* = 230) experienced no change or deterioration in “taste” function.

When extrapolated to national populations, persistent or worsening smell loss was estimated to affect 647,064 individuals in Austria (95% CI: 547,662–746,466), 6,221,556 in Germany (95% CI: 5,703,239–6,739,873), and 502,175 in Switzerland (95% CI: 348,950–655,399) (Table 3).

#### Prevalence comparison between nations

Pearson’s chi-square test indicated significant differences in the prevalence of parosmia (χ² = 5.975, df = 2, *p* = 0.050), phantosmia (χ² = 8.985, df = 2, *p* = 0.011), olfactory intolerance (χ² = 16.342, df = 2, *p* < 0.001), and “taste” impairment (χ² = 16.144, df = 2, *p* < 0.001) across countries. No statistically significant difference was observed in the prevalence of smell loss (χ² = 1.244, df = 2, *p* = 0.537).

Post hoc 2 × 2 chi-square comparisons revealed that phantosmia was significantly more prevalent in Switzerland compared to Austria (χ² = 7.943, df = 1, *p* = 0.005) and Germany (χ² = 7.770, df = 1, *p* = 0.005). Olfactory intolerance was also significantly higher in Switzerland than in Austria (χ² = 14.339, df = 1, *p* < 0.001) and Germany (χ² = 14.168, df = 1, *p* < 0.001). “Taste” impairment was more prevalent in Switzerland than in Austria (χ² = 16.200, df = 1, *p* < 0.001) and Germany (χ² = 8.300, df = 1, *p* < 0.001). No significant differences were observed between Austria and Germany across any of the SATDs domains.

#### Logistic regression

Multivariable logistic regression analysis identified several significant predictors of reporting SATDs. Compared to individuals aged 18–24 years, those aged 25–34 (OR = 0.36, 95% CI: 0.21–0.62, *p* < 0.001), 35–44 (OR = 0.45, 95% CI: 0.22–0.89, *p* = 0.023), 55–64 (OR = 0.54, 95% CI: 0.31–0.93, *p* = 0.027), and 65–74 (OR = 0.46, 95% CI: 0.28–0.77, *p* = 0.013) had significantly lower odds of reporting SATDs.

Male gender was associated with lower odds of reporting SATDs compared to females (OR = 0.63, 95% CI: 0.50–0.79, *p* < 0.001). Current smokers were significantly more likely to report a SATD than never smokers (OR = 1.59, 95% CI: 1.10–2.20, *p* = 0.009) (Table 4).

**Table 4:** Binomial Logistic Regression for Self-Reported SATDs

	OR	95% CI	*p*-value
Age			
18–24	—	—	—
25–34	0.360	0.21, 0.62	< 0.001
35–44	0.446	0.22, 0.89	0.023
45–54	0.516	0.24, 1.1	0·092
55–64	0.537	0.31, 0.93	0.027
65–74	0.464	0.28, 0.77	0.013
75 and over	0.615	0·32, 1.17	0.137
**Gender**			
Female	—	—	—
Male	0.629	0.50, 0.79	< 0·001
Non-binary	0.76	0.07, 8.50	0.824
**Tobacco smoking**			
Never Smoked	—	—	—
Current Smoker	1.59	1.1, 2.2	0.009
Ex-Smoker	1.16	0.83,1.62	0.383
**Alcohol consumption > 4 units/day**			
No	—	—	—
Yes	1.52	0.74, 3.09	0.252

*OR* Odds Ratio,*CI* Confidence Interval

#### Ordinal regression: Quality of life

An ordinal logistic regression model was used to examine associations between smell and taste disorders (SATDs) and self-reported quality of life (QoL). Reporting one or more SATDs was significantly associated with higher odds of poorer QoL (OR = 0.30, 95% CI: 0.24–0.38, *p* < 0.001, reference = symptom present). At the level of individual symptoms, participants without phantosmia (OR = 0.38, 95% CI: 0.28–0.51, *p* < 0.001), without parosmia (OR = 0.28, 95% CI: 0.15–0.49, *p* < 0.001), and without taste loss (OR = 0.61, 95% CI: 0.41–0.90, *p* = 0.013) had reported better QOL scores compared to those with these symptoms. By contrast, smell loss did not significantly differentiate QoL outcomes (OR = 1.04, 95% CI: 0.75–1.46, *p* = 0.804) (Table 5).

**Table 5:** Ordinal Logistic Regression of SATDs and QOL

		OR	*p*-value	95% CI
**Age**				
18–24		0.80	0.51	0.42, 1.53
25–34		0.93	0.89	0.35, 2.46
35–44		1.62	0.21	−1.04, 3.49
45–54		2.26	0.05	0.99, 5.17
55–64		0.96	0.89	0.51, 1.80
65–74		0.84	0.55	0.47, 1.80
75 and over		—	—	—
**Smell Domain**				
> 1 or more SATD (no vs. yes)		0.30	< 0.001	0.24, 0.38
Smell Loss (no vs. yes)		1.04	0.804	0.75, 1.46
Parosmia (no vs. yes)		0.28	< 0.001	0.15, 0.49
Phantosmia (no vs. yes)		0.38	< 0.001	0.28, 0.51
Olfactory Intolerance (no vs. yes)		1.88	0.032	1.06, 3.35
Taste Loss (no vs. yes)		0.61	0.013	0.41, 0.90
Gender				
Female		1.09	0.94	0.09, 13.00
Male		1.43	0.78	0.12, 17.02
Non-binary		—	—	—
Tobacco smoking				
Never Smoked		1.05	0.78	0.75, 1.47
Current Smoker		1.09	0.61	0.79, 1.50
Ex-Smoker		—	—	—
Alcohol consumption > 4 units/day				
No		1.18	0.26	0.88, 1.58
Yes		—	—	—
Country				
Austria		1.10	0.61	0.76, 1.60
Germany		1.04	0.85	0.71, 1.52
Switzerland		—	—	—

The proportional odds assumption for the ordinal regression model was met (Test of Parallel Lines, *p* > 0.05), supporting the appropriateness of the model. Pseudo R² values indicated modest explanatory power (Cox and Snell = 0.135; Nagelkerke = 0.152; McFadden = 0.066).

## Discussion

### Key results

Among 2,340 respondents, over 40% reported at least one SATD, with olfactory intolerance, phantosmia, and parosmia being most frequent. Between 48% and 62% of individuals experienced unchanged or worsening symptoms, highlighting a substantial burden of persistent dysfunction. Swiss respondents reported significantly higher rates of parosmia, phantosmia, olfactory intolerance, and taste impairment than Austrian or German participants, potentially reflecting differences in COVID-19 exposure and pandemic restrictions.

### Strengths and limitations

This study has several strengths. It is among the first large, cross-national assessments of SATDs in the general population after the COVID-19 pandemic, covering diverse demographic groups in Austria, Germany, and Switzerland. Use of the validated, cross-culturally adapted Smell-Qx questionnaire strengthens reliability and comparability [18]. A further strength is the focus on qualitative disorders, which are often overlooked in research and clinical care.

Several limitations should be noted. All data were self-reported, raising the possibility of recall and reporting bias, particularly regarding symptom onset and perceived causes. Older adults and individuals less comfortable with digital technology were less likely to complete the online survey, which may partly explain the lower prevalence of SATDs observed in these groups. Recruitment via an online market research panel may have introduced selection bias, and the subset of participants providing complete responses may have been subject to response bias, as those experiencing symptoms could have been more motivated to complete all survey items. Objective diagnostic testing (e.g., Sniffin’ Sticks) was not conducted, limiting confirmation of olfactory dysfunction. In addition, voluntary participation may have favoured individuals with persistent or severe symptoms, although these participants represent those likely most in need of support. Quality-of-life outcomes were derived from a single global item rather than domain-specific subscales; although the full Smell-Qx instrument captures granular domains (e.g., nutrition, safety, and mental health), these items were not completed consistently across respondents thereby preventing further analysis. Furthermore, prevalence estimates assume representativeness, which was assessed based on age, gender, and education. However, ethnicity data were not collected, limiting evaluation of disparities across subgroups.

Moreover, population-level extrapolations of SATDs prevalence were based on observed sample percentages applied to national population figures. These estimates assume uniform prevalence across the population and are derived from a non-probabilistic, self-reported sample; therefore, they should be interpreted as illustrative rather than definitive epidemiologic values. Quality of life was assessed with a validated tool, but only a single-item measure was used due to low response rates, restricting detail on specific domains. Our extrapolations to national populations assumed a uniform prevalence across all age groups. However, olfactory dysfunction is known to vary with age, and national age-specific data were not available to allow for adjustment.

### Interpretation

Qualitative olfactory symptoms (phantosmia, parosmia) were more prevalent than quantitative deficits such as anosmia or hyposmia and were strongly associated with reduced quality of life. This pattern aligns with the regenerative phase of olfactory recovery, during which miswiring of olfactory sensory neurons may lead to distorted perceptions [12, 14, 22]. This process can result in abnormal neural reconnections, contributing to distorted sensory perceptions [12, 14, 22]. The “miswiring hypothesis” proposes that during OSN regeneration, axons may mistarget their destinations or olfactory receptor expression may shift, leading to distorted odour processing. While parosmia is often considered a sign of ongoing recovery, phantosmia may reflect more entrenched or maladaptive regeneration [12, 23]. Prior studies have demonstrated that the prevalence of phantosmia tends to plateau around eight weeks post-infection and then persist beyond 60 weeks [24]. This pattern suggests that, in some individuals, olfactory regeneration may be prolonged or incomplete, contributing to persistent symptoms [22, 25].

Building on this clinical picture, geographic differences in symptom burden further inform our understanding and reflect broader epidemiological trends. Switzerland’s relatively more lenient public health restrictions during the pandemic, particularly in its early phases, possibly resulted in higher cumulative infection rates and, consequently, may have increased exposure to SARS-CoV-2 across the population [26, 27]. Greater viral exposure may explain the higher rates of olfactory and gustatory dysfunction in this subgroup. These findings also suggest a shift in the predominant causes of olfactory disorders in the region. Prior to the pandemic, data from Damm et al. (2004) estimated that approximately 79,000 patients per year were treated for olfactory disorders in hospitals across these three countries; primarily due to inflammatory sinonasal disease [10]. Reported aetiologies should be interpreted cautiously. All respondents who reported an identifiable cause attributed symptoms to post-infectious events, most commonly secondary to COVID-19. This likely reflects recall bias, heightened awareness of COVID-related olfactory dysfunction, and limited clinical evaluation rather than the true distribution of causes.

The impact of qualitative symptoms on patients’ lives is particularly concerning. Participants with parosmia and phantosmia reported significantly lower QoL scores, consistent with prior research linking these symptoms to depression, anxiety, and eating disturbances [28]. The Altered Eating Framework offers insight into this relationship, highlighting how distorted odour and taste perceptions interfere with food enjoyment, nutrition, and social interactions. In contrast, olfactory intolerance, though common, had a relatively smaller impact on QoL, potentially due to the ability of affected individuals to identify and avoid specific triggers [29].

The potential burden of SATDs highlights the need to develop effective treatment strategies. Currently, there is a lack of targeted therapies, and existing management approaches are largely based on expert consensus rather than high-quality evidence. A recent consensus statement acknowledged this gap and recommended that treatment for parosmia and phantosmia associated with quantitative disorders follow general protocols for post-infectious SATDs [12]. Experimental interventions, including vitamin A, omega-3 fatty acids, platelet-rich plasma, olfactory implants, and gene therapy, have shown early promise but require rigorous evaluation in well-designed clinical trials. There is also anecdotal evidence supporting the use of intranasal sodium citrate in reducing phantosmia, and case reports have suggested benefits from antimigraine, antipsychotic, or antiepileptic medications in selected patients, depending on the underlying aetiology [12]. However, these approaches are not yet standardised or supported by large-scale studies [12, 14].

A coordinated research agenda is needed. This should include robust clinical trials and studies that utilise the core outcome set developed by the Clinical Olfactory Working Group (COWoG), and also align with the top 10 research priorities outlined by the James Lind Alliance Priority Setting Partnership for SATDs [1, 2, 12, 30]. Moreover, a coordinated research agenda requires improved funding for SATDs, and the development of centralized referral pathways and multidisciplinary treatment frameworks. Without these, individuals with SATDs will potentially remain at risk of prolonged morbidity and poor quality of life.

### Generalizability

The large, cross-national sample and use of stratified quota sampling enhance the external validity of our findings, suggesting that the prevalence estimates are broadly representative of the general adult populations of Austria, Germany, and Switzerland. The use of a validated, cross-culturally adapted instrument further supports comparability across linguistic groups. However, reliance on self-reported data and the voluntary nature of participation may limit generalisability to populations less likely to engage in online surveys, such as older adults, individuals with lower digital literacy, or those outside high-income European settings.

## Conclusion

This multi-country study underscores the persistent burden of SATDs in the post-COVID era, with significant implications for both public health and clinical care. Symptoms such as parosmia and phantosmia remain prevalent long after infection and are strongly associated with reduced quality of life. As the epidemiology of SATDs shifts in the wake of the pandemic, there is a need to re-evaluate diagnostic frameworks, develop evidence-based treatment strategies, and implement structured, multidisciplinary care pathways. Addressing these sensory disorders must become a clinical and research priority to mitigate their long-term impact on patients’ wellbeing.

## Data Availability

Data is available upon reasonable request to the corresponding author.
